# Clinical Efficacy of Smartphone App–Based Pulmonary Rehabilitation in Chronic Respiratory Diseases: Randomized Controlled and Feasibility Trials

**DOI:** 10.2196/76801

**Published:** 2025-11-28

**Authors:** Chiwook Chung, Ah-Ram Kim, Do-Yoon Kang, Sunmok Kim, Jinyoung Oh, Hui Jung Kim, Byongjo Park, Seong Ho Lee, Dongbum Kim, Hee Kwon, Min-Woo Jo, Sei Won Lee

**Affiliations:** 1 Department of Pulmonary and Critical Care Medicine Asan Medical Center University of Ulsan College of Medicine Seoul Republic of Korea; 2 Division of Pulmonary, Allergy and Critical Care Medicine, Department of Internal Medicine Hallym University Dongtan Sacred Heart Hospital Hallym University College of Medicine Hwaseong Republic of Korea; 3 Department of Cardiology Asan Medical Center University of Ulsan College of Medicine Seoul Republic of Korea; 4 Maseok Asan Clinic Internal Medicine Namyangju Republic of Korea; 5 Seoul Comfort Medical Clinic Goyang Republic of Korea; 6 Good Breath Internal Medicine Clinic Gunpo Republic of Korea; 7 Seoul Eco Internal Medical Clinic Seoul Republic of Korea; 8 LifeSemantics Corp. Seoul Republic of Korea; 9 Department of Preventive Medicine Asan Medical Center University of Ulsan College of Medicine Seoul Republic of Korea

**Keywords:** exercise capacity, physical activity, pulmonary rehabilitation, quality of life, mHealth, digital health

## Abstract

**Background:**

Pulmonary rehabilitation improves exercise capacity, dyspnea symptoms, quality of life, and even survival in patients with chronic respiratory disease. Center-based pulmonary rehabilitation programs often face barriers, and alternatives to center-based rehabilitation are urgently needed.

**Objective:**

This study aimed to evaluate the clinical efficacy of smartphone app–based pulmonary rehabilitation in patients with chronic respiratory diseases, including chronic obstructive pulmonary disease, interstitial lung disease, and bronchiectasis.

**Methods:**

This randomized controlled trial recruited 90 participants, randomly allocated into intervention (n=60) and control (n=30) groups. The intervention group received a 12-week smartphone app–based rehabilitation program, while the control group received standard outpatient care. The primary outcomes were maximal oxygen consumption via cardiopulmonary exercise test and the chronic obstructive pulmonary disease assessment test (CAT) after a 12-week period. Based on changes in quality-of-life questionnaire index scores, quality-adjusted life years were calculated, and a cost-utility analysis was conducted. A feasibility trial was also conducted in 4 community primary health care clinics.

**Results:**

Of the 70 participants (median age 65.5 years) who completed the follow-up visits, 67 were included in the per-protocol analysis. The intervention group (n=43) showed significant improvements compared with the control group (n=24) in CAT score (median 7.0, IQR 4.0-15.0 vs median 10.0, IQR 6.5-18.5; *P*=.04), and International Physical Activity Questionnaire score (median 1488.0, IQR 1250.3-3027.8 vs median 1164.0, IQR 618.8-2205.0; *P*=.04), but not maximal oxygen consumption. Clinical outcomes showed more prominent improvements among participants who were physically active or compliant with rehabilitation programs. In the user experience survey, around 80% (35/43) of participants in the intervention group found the app easy to use, and more than 60% (27/43) reported that it helped improve dyspnea symptoms. The mean total health care costs were US $495 in the control and US $523 in the intervention group, with no notable difference in the quality-adjusted life year distribution. In the feasibility trial, 24 participants completed follow-up visits, showing a significant reduction in CAT score (median 8.5, IQR 6.0-18.0 to median 5.0, IQR 2.0-7.5; *P*<.001) post rehabilitation. No participants experienced disease exacerbation or musculoskeletal injury related to the rehabilitation activities.

**Conclusions:**

The randomized controlled trial demonstrated that a smartphone-based pulmonary rehabilitation program improved clinical outcomes, including quality of life, physical activity, and dyspnea, in patients with chronic respiratory diseases. Although physically active and program-compliant participants showed significant clinical improvements, the fact that less than half of the participants demonstrated good compliance warrants more robust strategies to enhance adherence in future programs. Additionally, our feasibility trial demonstrated the potential for rehabilitation programs for older adults with chronic respiratory diseases to be implemented in primary health care settings. This approach represents a novel, scalable model bridging hospital- and community-based care, demonstrating real-world feasibility for digital rehabilitation in chronic respiratory diseases.

**Trial Registration:**

ClinicalTrials.gov NCT05610358; https://clinicaltrials.gov/ct2/show/NCT05610358

## Introduction

Chronic respiratory diseases are major causes of global morbidity and mortality [1,2]. In 2019, chronic obstructive pulmonary disease (COPD), lung cancer, and lower respiratory infections ranked among the top 10 causes of disability-adjusted life-years for individuals older than 50 years [1]. Moreover, those with chronic respiratory diseases face additional challenges, including reduced quality of life and limited physical activity and exercise capacity [3,4].

Pulmonary rehabilitation offers a comprehensive approach to improving physical and psychological well-being in people with chronic respiratory disease, incorporating exercise, behavior change, and education [4]. It has been shown to effectively enhance exercise capacity, quality of life, dyspnea, and even survival rates, especially in patients with COPD [3-5]. Muscle wasting and dysfunction, affecting both skeletal and respiratory muscles, are also common in these patients, alongside reduced physical activity and respiratory function [6,7]. Pulmonary rehabilitation, which includes intensive exercise training and nutritional support, may be the most effective intervention for such conditions [7].

Landmark studies have recommended pulmonary rehabilitation programs with exercise training sessions lasting 30-45 minutes per day, 3-5 days per week, for at least 8-12 weeks [8,9]. However, center-based programs often face barriers such as limited facilities, low awareness among health care providers, lack of patient motivation, minimal health insurance cost and social support, and transport challenges [10-12]. Moreover, it is also challenging for patients with chronic respiratory disease to maintain the rehabilitation programs in daily lives, as they did not receive sufficient instructions to continue their programs at home, resulting in insufficient participation and high dropout rates in center-based pulmonary rehabilitation [4,13,14]. Consequently, alternatives to center-based rehabilitation are urgently needed [15-22]. The COVID-19 pandemic further accelerated the development of telerehabilitation alternatives to center-based pulmonary rehabilitation [23]. Thus, researchers have investigated the clinical efficacy of telerehabilitation compared with traditional face-to-face rehabilitation in individuals with COPD [15-22].

Although telerehabilitation has been proposed previously, it encompasses several different models, including video conferencing, mobile apps, webpages, and audio or video telephone calls [24]. Among telerehabilitation modalities, the clinical evidence for smartphone app–based pulmonary rehabilitation remains inconclusive [25]. While such interventions have been shown to improve quality of life and alleviate respiratory symptoms, their effect on enhancing exercise capacity has been limited [25-27]. Notably, significant improvements in exercise capacity have been observed only among users with high adherence [27]. However, recent studies suggest that app-based rehabilitation programs are effective not only in maintaining physical activity but also in improving exercise capacity, highlighting their potential for future implementation in clinical practice [28,29]. Furthermore, pilot studies have suggested the potential of reducing hospital readmissions after COPD exacerbations compared with standard care [30].

In previous work, we reported positive outcomes in patients with chronic respiratory disease using a smartphone-based rehabilitation program in a single-arm prospective study [31]. In this study, we assessed the clinical efficacy of smartphone-based rehabilitation through a randomized controlled trial, with the study protocol being previously published [32]. Additionally, we conducted a feasibility trial (a single-arm prospective study) across 4 community primary health care clinics to evaluate whether an app-based rehabilitation program is feasible in primary health care facilities with limited medical resources. This study aimed to evaluate the clinical efficacy of smartphone app–based pulmonary rehabilitation programs in improving exercise capacity, quality of life, and respiratory symptoms in individuals with chronic respiratory diseases.

## Methods

### Study Design

This study adhered to the CONSORT statement (Multimedia Appendix 1) [33]. This single-center, single-blind randomized controlled trial was conducted at Asan Medical Center in Seoul, Republic of Korea. In 2023, a total of 90 participants were recruited from the pulmonology outpatient clinic and randomly assigned to the intervention or control group in a 2:1 ratio (60 in the intervention group and 30 in the control group). This 2:1 allocation strategy ensured that a larger number of participants were assigned to the intervention group, which helped maintain statistical power despite the potential for higher dropout rates in that group and allowed for more robust subgroup analyses. The initial plan was to include 81 participants (54 in the intervention group and 27 in the control group), based on an estimation for detecting significant improvement in maximal oxygen consumption (VO_2_ max) [32]; however, due to a higher-than-expected dropout rate, we ultimately enrolled 90 participants.

The intervention lasted 12 weeks. The intervention group underwent app-based rehabilitation for 12 weeks. They received one protein supplement drink (Himmune Protein Balance Drink, 10 g protein per 190 mL pack, Ildong Foodis, Seoul, Republic of Korea) daily throughout the study period, as adequate protein intake was required during the rehabilitation program [34]. The control group received standard outpatient medical treatment without rehabilitation. Participants were assessed at baseline and again at the end of the 12-week rehabilitation period.

A subsequent feasibility trial was conducted in 4 community primary health care clinics (Maseok Asan Clinic Internal Medicine, Seoul Comfort Medical Clinic, Good Breath Internal Medicine Clinic, and Seoul Eco Internal Medical Clinic). In 2023, 32 participants (different from the original randomized controlled trial participants) were recruited and underwent the 12-week app-based rehabilitation. They were assessed twice: at baseline and at the end of the program.

### Inclusion and Exclusion Criteria

The inclusion criteria were as follows: (1) age of 20-80 years; (2) a dyspnea symptom score of ≥1 on the modified Medical Research Council (mMRC) dyspnea scale; and (3) a chronic respiratory disease, including (1) obstructive lung disease (such as asthma and COPD) with a forced expiratory volume in one second (FEV_1_) of <80% of the predicted value or FEV_1_/forced vital capacity (FVC) of <0.7, (2) bronchiectasis visible in more than one lung lobe on chest computed tomography, or (3) restrictive lung disease (such as tuberculous lung destruction or interstitial lung disease) with FVC or diffusing capacity for carbon monoxide (DL_CO_) of <80% of the predicted value [35].

The exclusion criteria were (1) disease exacerbation within 4 weeks before enrolment, (2) disabilities, (3) inability to use smartphone apps or being an iPhone user, (4) pregnancy or breastfeeding, (5) deemed unsuitable for participation by the attending physician, and (6) refusal to consent.

### Smartphone App and Rehabilitation Program

The SENIORS smartphone app (LifeSemantics Corp, Seoul, Republic of Korea) was developed through collaboration with health care professionals, service designers, and software developer. It was built for Android devices (requiring Android 8.0 or later).

The app included features such as exercise programs, exercise records, exercise partners, a 6-minute walk test, disease education specific to each respiratory disease, inhaler usage guidance, and a medication and health data diary. The pulmonary rehabilitation program consisted of 30 minutes of aerobic exercise (outdoor walking) and 20-30 minutes of limb and trunk muscle exercise. Smartphone sensors automatically track walking exercises without additional pedometers. The muscle exercise included warm-up stretching, a main exercise of varied muscle workouts with progressively increasing intensity (weekly), and cool-down stretching. Participants engaged in the rehabilitation program independently using the app, without intervention from the center site. The app displayed daily pop-up messages to encourage continued participation. Participants could earn incentives based on their exercise records (Figure S1 in Multimedia Appendix 2).

### Study Outcome

The primary outcomes were VO_2_ max measured by cardiopulmonary exercise test [36], and the COPD assessment test (CAT) [37]. Cardiopulmonary exercise test was conducted using an incremental protocol with a cycle ergometer (VIAsprint 150P; Carefusion) and a metabolic cart (Vmax 29; SensorMedics) [36]. To assess long-term rehabilitation effects, CAT scores for the intervention group were re-evaluated at 12 weeks post rehabilitation (24 weeks from baseline). The app was unavailable during the postrehabilitation period.

Secondary outcomes included daily physical activity measured by the International Physical Activity Questionnaire Short Form (IPAQ) Korean Version [38], mMRC dyspnea scale, quality of life questionnaires, such as EQ-5D-5L [39,40] and the Health-related Quality of Life Instrument with 8 Items (HINT-8) questionnaire [41], lung function (FVC, FEV_1_, and DL_CO_), hand grip strength, and limb muscle mass measured by bioelectrical impedance analysis.

The study outcomes were simplified in a feasibility study conducted in primary health care clinics. Primary outcomes were the 6-minute walk test distance and CAT [42], and secondary outcomes included lung function (FVC and FEV_1_).

### Cost-Utility Analysis

The main outcome indicators for the cost-utility analysis were the EQ-5D-5L and HINT-8 indexes, measured at baseline and the end of the intervention. Quality-adjusted life year (QALY) was calculated as the change in EQ-5D-5L or HINT-8 index from baseline to the end of the intervention for each participant [43]. Health care costs related to chronic respiratory diseases were gathered from invoices issued by Asan Medical Center, and outpatient drug costs were calculated based on prescription history. Based on the subscription costs of existing commercial applications, the app subscription fee in the intervention group was set at US $40 per month (approximately US $1=1400 Korean won as of November 2024), totaling US $120 for 3 months, with participant incentives deducted from this amount.

### Statistical Analysis

Continuous variables were presented as medians with IQRs and compared using the Mann-Whitney *U* test (to compare the intervention and control groups at the same time point) or Wilcoxon signed-rank test (to compare baseline and follow-up within each group), as the number of participants was relatively small and most variables did not follow a normal distribution. A paired *t* test was conducted to evaluate the mean differences and corresponding CIs of normally distributed continuous variables (the CAT score and EQ-5D-5L index) before and after the intervention. Categorical variables were reported as numbers (percentages) and compared using the chi-square or Fisher exact test. All *P* values were 2-tailed, with statistical significance set at *P*<.05. All statistical analyses were conducted using SPSS (version 26.0; IBM SPSS Corp) and MedCalc Statistical Software (version 23.0.5; MedCalc Software Ltd).

### Ethical Considerations

The study protocol was approved by the Institutional Review Board of Asan Medical Center (2022-1460) and the Public Institutional Review Board designated by the Ministry of Health and Welfare (P01-202303-01-013). This study adhered to the Declaration of Helsinki guidelines, and all procedures were conducted in compliance with relevant regulations. It was prospectively registered in the ClinicalTrials.gov database (NCT05610358). Written informed consent was obtained from all participants before inclusion. Participants were informed that their participation was voluntary and that they could withdraw from the study at any time without any disadvantage or penalty. All identifiable data were removed from the original dataset, and each participant was anonymized. Participants received small incentives (approximately US $30) based on their exercise records, which were obtained from the app’s log data for aerobic exercise timers and anaerobic exercise instruction videos. No identification of individual participants is possible in any images included in the manuscript or in Multimedia Appendices.

## Results

### Study Process and Baseline Characteristics

**Figure 1:**
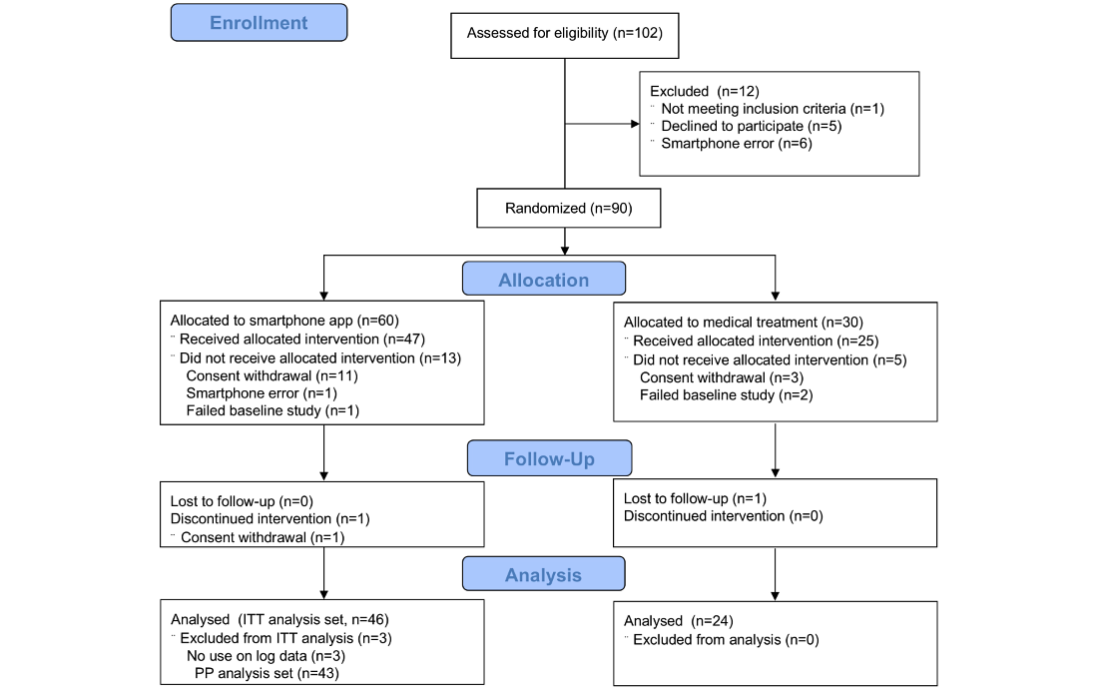
Study flowchart of a randomized controlled trial investigating the clinical efficacy of smartphone-based rehabilitation in individuals with chronic respiratory disease, conducted at Asan Medical Center, Seoul, Republic of Korea, 2023. ITT: intention to treat; PP: per protocol.

Figure 1 shows the study process. Among 102 screened for eligibility, 90 participants were recruited and allocated to the intervention (n=60) and control (n=30) groups. Thereafter, 14 participants withdrew consents, one encountered a smartphone error, and 3 failed baseline studies. Consequently, 72 participants completed baseline assessments. In the intervention group, 47 participants began rehabilitation, and 46 completed follow-ups (included in the intention-to-treat analysis). Three were later identified as having no app-use history in log data, so 43 were included in the per-protocol analysis. In the control group, 25 began follow-up, with 24 completing it. Overall, 77.8% (70/90) of participants completed follow-ups, and no musculoskeletal injuries or disease exacerbations related to the rehabilitation program were reported.

Table 1 presents the baseline characteristics of 70 participants who completed follow-ups. The median age was 65.5 years (IQR 61.0-72.0), with 48 (68.6%) men. Forty-four (62.9%) participants had a smoking history, and approximately three-quarters (53/70, 75.7%) had obstructive lung disease as their chronic respiratory disease.

**Table 1:** Baseline characteristics of participants in a randomized controlled trial investigating the clinical efficacy of smartphone-based rehabilitation in individuals with chronic respiratory disease, conducted at Asan Medical Center, Seoul, Republic of Korea, 2023.

		Total (n=70)	Intervention (n=46)	Control (n=24)	*P* value		*U* value	
Age (years), median (IQR)		65.5 (61.0-72.0)	64.0 (60.0-68.0)	67.5 (62.5-74.5)	.14		434.00	
Sex (male), n (%)		48 (68.6)	35 (76.1)	13 (54.2)	.06		N/A^a^	
Body weight (kg), median (IQR)		61.0 (54.0-71.4)	61.0 (54.0-71.8)	59.0 (53.0-70.2)	.34		483.50	
Height (cm), median (IQR)		167.0 (160.0-172.0)	168.1 (161.0-172.0)	164.5 (157.0-168.0)	.04		383.00	
BMI (kg/m^2^), median (IQR)		23.0 (20.0-25.4)	23.0 (19.8-25.3)	22.9 (20.8-25.6)	.89		541.00	
Ever-smoker		44 (62.9)	32 (69.6)	12 (50.0)	.11		N/A	
**Respiratory disease, n (%)**						.29		N/A
	Obstructive	53 (75.7)	34 (73.9)	19 (79.2)				
	Bronchiectasis	14 (20.0)	11 (23.9)	3 (12.5)				
	Restrictive	3 (4.3)	1 (2.2)	2 (8.3)				
**Comorbidities, n (%)**								
	Diabetes mellitus	9 (12.9)	5 (10.9)	4 (16.7)	.48		N/A	
	Hypertension	20 (28.6)	13 (28.3)	7 (29.2)	.94		N/A	
	Dyslipidemia	18 (25.7)	11 (23.9)	7 (29.2)	.64		N/A	
	Malignancy	6 (8.6)	2 (4.3)	4 (16.7)	.17		N/A	
	Lung resection	3 (4.3)	1 (2.2)	2 (8.3)	.27		N/A	

^a^N/A: not applicable.

### Clinical Parameters of Participants

In the per-protocol analysis, the baseline CAT score was higher in the intervention group than in the control group (median 16.0 vs 11.0, *P*=.08). At follow-up, the intervention group had a significantly lower CAT score than the control group (median 7.0 vs 10.0, *P*=.04). VO_2_ max did not significantly differ between groups at follow-up (median 14.0 vs 12.7 mL/kg/min, *P*=.24). The intervention group also showed significant improvements in the IPAQ score (median 1488.0 vs 1164.0, *P*=.04) and mMRC dyspnea scale (median 1.0 vs 2.0, *P*=.01) compared with the control group at follow-up (Table 2). In the intention-to-treat analysis, only the mMRC dyspnea scale showed a significant difference at follow-up, with no significant changes in the CAT score and IPAQ (Table S1 in Multimedia Appendix 2).

Comparing clinical outcomes between baseline and follow-up in the intervention group, CAT score, IPAQ, mMRC dyspnea scale, EQ-5D-5L index, and HINT-8 index showed significant improvements, while these parameters, except for IPAQ, did not improve in the control group (Table 3 and Table S2 in Multimedia Appendix 2). In the intervention group, the CAT score showed a significant improvement from baseline to post-intervention (mean difference=–6.14, 95% CI –8.19 to –4.09, *P*<.001, t_42_=–6.051), exceeding the minimal important clinical difference of 2 points [44]. The EQ-5D-5L index demonstrated a meaningful increase (mean difference=0.073, 95% CI 0.042-0.103, *P*<.001, t_42_=4.814), surpassing the minimal important clinical difference for COPD (0.028) [45]. To assess long-term rehabilitation effects, the CAT score in the intervention group remained stable 12 weeks post-rehabilitation, showing no significant difference from the score at the end of rehabilitation (median 7.0 vs 8.0, *P*=.77, Figure S2 in Multimedia Appendix 2).

**Table 2:** Comparison of clinical outcomes of participants between the intervention and control group (per protocol analysis) in a randomized controlled trial investigating the clinical efficacy of smartphone-based rehabilitation in individuals with chronic respiratory disease, conducted at Asan Medical Center, Seoul, Republic of Korea, 2023.

			Total (n=67), median (IQR)	Intervention (n=43), median (IQR)	Control (n=24)	*P* value	*U* value
**Baseline**							
	VO_2_ max^a^ (mL/kg/min, n=65)		15.3 (11.5-18.3)	15.7 (12.7-19.2)	13.4 (9.0-16.1)	.06	339.50
	CAT^b^ score		14.0 (8.0-19.0)	16.0 (9.5-20.0)	11.0 (7.5-17.0)	.08	380.50
	IPAQ^c^ (n=65)		693.0 (23.1-1535.3)	792.0 (26.0-1737.8)	445.5 (11.6-1188.0)	.23	386.50
	mMRC^d^ dyspnea scale		1.0 (1.0-2.0)	1.0 (1.0-2.0)	2.0 (1.0-2.0)	.53	472.50
	EQ-5D-5L index		0.816 (0.752-0.862)	0.816 (0.743-0.861)	0.822 (0.777-1.000)	.41	453.50
	HINT-8^e^ index		0.804 (0.750-0.862)	0.792 (0.735-0.859)	0.811 (0.784-0.863)	.16	409.00
	FEV_1_^f^ (%predicted)		51.0 (42.5-65.8)	57.0 (44.3-66.8)	49.0 (38.5-56.0)	.11	394.00
	FVC^g^ (%predicted)		73.0 (66.3-87.5)	75.0 (66.3-87.5)	72.0 (64.0-86.0)	.39	450.00
	DL_CO_^h^ (%predicted)		58.0 (45.0-67.0)	61.0 (53.5-67.0)	50.0 (45.0-65.0)	.30	436.00
	Hand grip strength (kg)		32.0 (25.7-39.2)	33.7 (28.0-41.0)	27.9 (21.5-37.1)	.12	397.50
	**Limb muscle mass (kg)**						
		Upper limb	4.8 (3.5-5.7)	5.0 (3.9-6.0)	4.3 (3.3-5.5)	.06	371.00
		Lower limb	15.0 (12.4-16.8)	15.6 (13.1-17.0)	14.5 (10.8-15.6)	.05	365.50
**Follow-up**							
	VO_2_ max (mL/kg/min, n=65)		12.9 (11.0-16.1)	14.0 (10.6-18.4)	12.7 (11.3-13.7)	.24	389.00
	CAT score		8.0 (5.0-16.8)	7.0 (4.0-15.0)	10.0 (6.5-18.5)	.04	358.50
	IPAQ (n=62)		1386.0 (876.0-2772.0)	1488.0 (1250.3-3027.8)	1164.0 (618.8-2205.0)	.04	290.50
	mMRC dyspnea scale		1.0 (1.0-2.0)	1.0 (1.0-1.8)	2.0 (1.0-2.0)	.01	346.50
	EQ-5D-5L index		0.862 (0.786-1.000)	0.871 (0.814-1.000)	0.829 (0.768-1.000)	.22	425.50
	HINT-8 index		0.821 (0.751-0.876)	0.828 (0.767-0.891)	0.795 (0.728-0.848)	.09	385.50
	FEV_1_ (%predicted)		50.0 (40.5-67.8)	58.0 (43.5-69.0)	45.0 (37.5-54.5)	.06	372.00
	FVC (%predicted)		73.0 (67.3-87.5)	77.0 (69.3-87.5)	71.5 (63.0-86.0)	.22	422.50
	DL_CO_ (%predicted, n=66)		61.0 (49.0-71.0)	62.0 (51.0-71.8)	57.0 (48.0-64.8)	.20	400.00
	Hand grip strength (kg)		32.7 (22.7-39.5)	33.3 (27.7-41.7)	24.0 (20.6-37.0)	.08	380.00
	**Limb muscle mass (kg)**						
		Upper limb	4.6 (3.6-5.9)	4.8 (3.8-6.1)	4.3 (3.5-5.5)	.17	411.50
		Lower limb	15.0 (12.2-16.8)	15.4 (13.0-16.9)	14.4 (10.8-16.0)	.08	381.00

^a^VO_2_ max: maximal oxygen consumption. It was measured using a cardiopulmonary exercise test.

^b^CAT: chronic obstructive pulmonary disease assessment test.

^c^IPAQ: International Physical Activity Questionnaire.

^d^mMRC: modified Medical Research Council.

^e^HINT-8: Health-related Quality of Life Instrument with 8 Items.

^f^FEV_1_: forced expiratory volume in one second.

^g^FVC: forced vital capacity.

^h^DL_CO_: diffusing capacity for carbon monoxide.

**Table 3:** Comparison of clinical outcomes of participants between the baseline and follow-up (per protocol analysis) in a randomized controlled trial investigating the clinical efficacy of smartphone-based rehabilitation in individuals with chronic respiratory disease, conducted at Asan Medical Center, Seoul, Republic of Korea, 2023.

			Baseline, median (IQR)	Follow-up, median (IQR)	*P* value
**Intervention (n=43)**					
	VO_2_ max^a^ (mL/kg/min)		15.7 (12.7-19.2)	14.0 (10.6-18.4)	.04
	CAT^b^ score		16.0 (9.5-20.0)	7.0 (4.0-15.0)	<.001
	IPAQ^c^ (n=41)		792.0 (23.1-1649.3)	1488.0 (1250.3-3027.8)	<.001
	mMRC^d^ dyspnea scale		1.0 (1.0-2.0)	1.0 (1.0-1.8)	.006
	EQ-5D-5L index		0.816 (0.743-0.861)	0.871 (0.814-1.000)	<.001
	HINT-8^e^ index		0.792 (0.735-0.859)	0.828 (0.767-0.891)	<.001
	FEV_1_^f^ (%predicted)		57.0 (44.3-66.8)	58.0 (43.5-69.0)	.27
	FVC^g^ (%predicted)		75.0 (66.3-87.5)	77.0 (69.3-87.5)	.04
	DL_CO_^h^ (%predicted)		61.0 (53.5-67.0)	62.0 (51.0-71.8)	.08
	Hand grip strength (kg)		33.7 (28.0-41.0)	33.3 (27.7-41.7)	.74
	**Limb muscle mass (kg)**				
		Upper limb	5.0 (3.9-6.0)	4.8 (3.8-6.1)	.47
		Lower limb	15.6 (13.1-17.0)	15.4 (13.0-16.9)	.56
**Control (n=24)**					
	VO_2_ max (mL/kg/min, n=22)		13.4 (9.0-16.1)	12.7 (11.3-13.7)	.45
	CAT score		11.0 (7.5-17.0)	10.0 (6.5-18.5)	.78
	IPAQ (n=21)		198.0 (11.6-1237.5)	1164.0 (618.8-2205.0)	.005
	mMRC dyspnea scale		2.0 (1.0-2.0)	2.0 (1.0-2.0)	.74
	EQ-5D-5L index		0.822 (0.777-1.000)	0.829 (0.768-1.000)	.26
	HINT-8 index		0.811 (0.784-0.863)	0.795 (0.728-0.848)	.08
	FEV_1_ (%predicted)		49.0 (38.5-56.0)	45.0 (37.5-54.5)	.46
	FVC (%predicted)		72.0 (64.0-86.0)	71.5 (63.0-86.0)	.78
	DL_CO_ (%predicted, n=23)		50.0 (45.0-65.0)	57.0 (48.0-64.8)	.17
	Hand grip strength (kg)		27.9 (21.5-37.1)	24.0 (20.6-37.0)	.29
	**Limb muscle mass (kg)**				
		Upper limb	4.3 (3.3-5.5)	4.3 (3.5-5.5)	.12
		Lower limb	14.5 (10.8-15.6)	14.4 (10.8-16.0)	.67

^a^VO_2_ max: maximal oxygen consumption. It was measured using a cardiopulmonary exercise test.

^b^CAT: chronic obstructive pulmonary disease assessment test.

^c^IPAQ: International Physical Activity Questionnaire.

^d^mMRC: modified Medical Research Council.

^e^HINT-8: Health-related Quality of Life Instrument with 8 Items.

^f^FEV_1_: forced expiratory volume in one second.

^g^FVC: forced vital capacity.

^h^DL_CO_: diffusing capacity for carbon monoxide.

The IPAQ questionnaire was administered during the cardiopulmonary exercise test, so these values were missing for the 2 participants with severely impaired lung function. Although some missing data (IPAQ and DL_CO_) were observed at the follow-up time point, the Little’s Missing Completely At Random test indicated that these data were missing completely at random (*P*=.12).

### Comparison of Clinical Parameters Among Subgroup Participants

**Figure 2:**
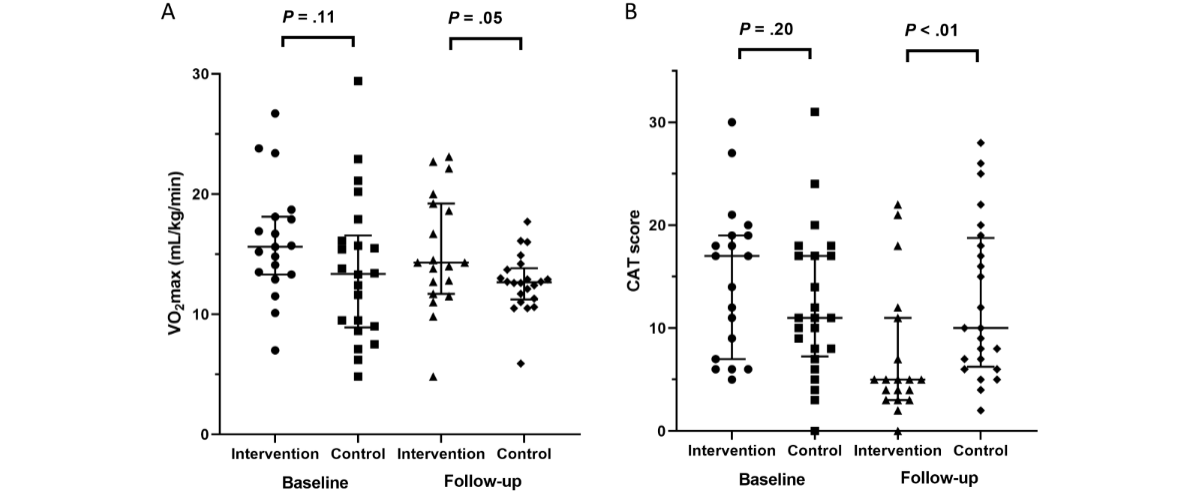
Subgroup analysis: comparison of primary outcomes between participants in the intervention group (initially active, International Physical Activity Questionnaire > 1000) and the control group in a randomized controlled trial investigating the clinical efficacy of smartphone-based rehabilitation in individuals with chronic respiratory disease, conducted at Asan Medical Center, Seoul, Republic of Korea, 2023. Data are shown for (A) maximal oxygen consumption and (B) CAT score. CAT: chronic obstructive pulmonary disease assessment test; VO2 max: maximal oxygen consumption.

Defining physically active participants as those with an IPAQ score of >1000 at baseline, 19 participants in the intervention group were classified as physically active. At follow-up, their VO_2_ max (median 14.3 vs 12.7 mL/kg/min, *P*=.05) and IPAQ (median 2779.0 vs 1164.0, *P*=.009) were significantly higher than those of the participants in the control group. Their CAT score (median 5.0 vs 10.0, *P*=.005) and mMRC dyspnea scale (median 1.0 vs 2.0, *P*=.03) were also significantly lower than those of the participants in the control group. Comparing baseline and follow-up data, physically active participants in the intervention group showed significant improvements in the CAT score, EQ-5D-5L index, and HINT-8 index (Tables S3 and S4 in Multimedia Appendix 2, Figure 2).

Defining good compliance as completing > 50% of the assigned exercise program, 17 participants in the intervention group were classified as compliant. At baseline, their CAT scores were significantly higher than those of the participants in the control group. At follow-up, their CAT score did not significantly differ from that of the control group, but their IPAQ was significantly higher. Comparing baseline and follow-up data, compliant participants in the intervention group showed significant improvements in CAT score, EQ-5D-5L index, and HINT-8 index after the intervention (Tables S5 and S6 in Multimedia Appendix 2).

### Ease of Use of the App

Among the user questionnaire of 43 participants who completed the rehabilitation program, approximately 80% (35/43) of participants in the intervention group found the app easy to use. More than 60% (27/43) felt it helped improve dyspnea symptoms. Additionally, 79.1% (34/43) expressed interest in using the app commercially, with 44.1% (15/34) noting that the exercise program tailored to their condition was its most attractive feature (Table S7 in Multimedia Appendix 2).

### Cost-Utility Analysis

**Figure 3:**
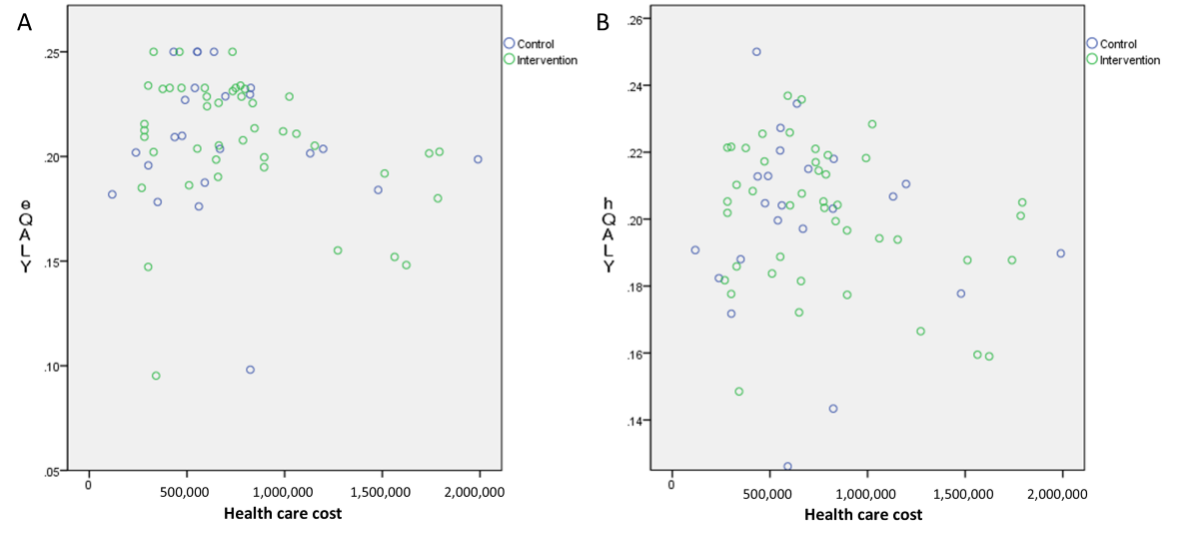
Cost-utility analysis: distribution of total health care costs and quality-of-life utility between the intervention and control groups in a randomized controlled trial investigating the clinical efficacy of smartphone-based rehabilitation in individuals with chronic respiratory disease, conducted at Asan Medical Center, Seoul, Republic of Korea, 2023. Data are shown for (A) Euro-QoL 5-Dimension 5-Level index and (B) HINT-8 index. eQALY: the quality-adjusted life year calculated using the EQ-5D-5L; hQALY, the quality-adjusted life year calculated using the HINT-8 index.

The mean hospital costs were US $300 for the control and US $250 for the intervention group. Outpatient drug costs averaged US $195 and US $189, respectively, while the app subscription fee averaged US $84 after incentive reductions. The mean total health care costs, including hospital, drug, and app fees, were US $495 in the control and US $523 in the intervention group. The QALY distribution using the EQ-5D-5L index was 0.208 for both groups, and using the HINT-8 index, it was 0.199 in the control and 0.201 in the intervention group (Figure 3).

### Feasibility Trial in Primary Health Care Clinics

Subsequently, a feasibility trial was performed in primary health care clinics. Among 32 participants initially recruited, 24 completed follow-up visits (Table S8 in Multimedia Appendix 2). The median age was 62.5 years (IQR 57.0-69.0), with 58.3% (14/24) being men and 54.2% (13/24) having a smoking history (Table S9 in Multimedia Appendix 2). Comparing baseline to follow-up data, the CAT score significantly improved post rehabilitation (median 8.5 to 5.0, *P*<.001, Table S10 in Multimedia Appendix 2). No musculoskeletal injuries or disease exacerbations related to the rehabilitation program were reported.

## Discussion

### Principal Findings

This study examined the clinical efficacy of a smartphone-based pulmonary rehabilitation program for patients with chronic respiratory diseases. It showed that the program improved the quality of life, daily physical activity, and dyspnea symptoms. Significant improvements were particularly notable in participants who were physically active or compliant with the exercise regimen, and positive outcomes were also observed in primary health care clinics. These findings suggest that smartphone-based pulmonary rehabilitation could be a valuable treatment option for older patients with chronic respiratory diseases.

### Improvements in Study Outcomes

This program was associated with a significant improvement in the CAT score, which predicts the severity of airflow obstruction and disease exacerbation in patients with COPD [46,47]. The CAT score also correlates with lung function, 6-minute walk distance, and exertional desaturation in idiopathic pulmonary fibrosis and connective tissue disease-associated interstitial lung disease [48,49]. CAT also correlated with disease severity, lung function, and 6-minute walk distance in patients with bronchiectasis, making it a valid tool for this condition [50]. Although we included patients with a broad spectrum of chronic respiratory diseases, CAT score improvements may broadly predict clinical benefits. Notably, CAT scores in the intervention group remained stable 12 weeks post-rehabilitation, further supporting the program’s efficacy.

Daily physical activity is crucial for patients with chronic respiratory disease, as low activity levels are linked to higher risks of acute exacerbation and mortality in COPD [51]. Patients with COPD generally have significantly reduced physical activity compared with healthy controls, though disease severity is not strongly correlated with activity levels [52]. Thus, even patients with severe COPD who stay active can reduce their exacerbation and mortality risks. This study found that physical activity improved through app-based pulmonary rehabilitation in older adults with chronic respiratory disease. Additionally, subgroup analysis showed marked clinical improvements in initially active participants, highlighting the importance of physical activity. Further studies with longer follow-ups could reveal reduced exacerbation and mortality rates from app-based rehabilitation.

### Compliance With Rehabilitation Programs

As reported, compliance with home-based pulmonary rehabilitation remains a challenge [53]. In app-based rehabilitation, CAT scores were estimated to decrease by −0.22 (95% CI −0.74 to 0.31) per additional 7 days of app use [54]. However, while app use dropped from 85% in week one to 40% in the last week, active users maintained a consistent weekly usage of 4.9 days [30]. A previous study reported lack of motivation as the most common reason for nonadherence in home-based pulmonary rehabilitation, with non-adherent patients with COPD experiencing more exacerbations and reduced 6-minute walk distances [53]. In this study, 39.5% (17 of 43) of participants showed good compliance (completing >50% of assigned exercises) and demonstrated significant improvements in CAT scores and IPAQ. Thus, physicians should emphasize steady engagement with the app-based pulmonary rehabilitation program. The initial stage of pulmonary rehabilitation involves educating patients on how following an appropriate exercise program can improve their symptoms. Additionally, regular phone calls or text messages from health care providers can boost patient motivation [55]. Repeated exposure to exercise programs encourages app use and motivates patients to stay active.

### Cost-Utility Analysis

In the cost-utility analysis, the mean health care costs were US $495 in the control group and US $523 in the intervention group. After reducing the app subscription fee (mean US $84 with incentive reductions), intervention group costs were US $439, notably lower than the control group. However, since the subscription fee was based on similar commercialized apps, it may be adjustable for future commercialization. While there was some cost difference between groups, QALYs based on EQ-5D-5L and HINT-8 indices were similar, limiting further cost-utility analysis. Previous cost-utility analyses have demonstrated that home-based pulmonary rehabilitation and tele-rehabilitation are more cost-effective compared with hospital-based pulmonary rehabilitation [16,56]. However, further studies are needed to evaluate the cost-effectiveness of these programs within the Korean health care context.

### Feasibility in Primary Clinics

Pulmonary rehabilitation is typically provided in referral hospitals; however, primary care practitioners face barriers in referring patients with chronic respiratory diseases, including limited engagement from rehabilitation providers, patient’s poor physical ability or accessibility, low motivation, and unclear referral practices [57]. To address these issues, previous studies have delivered rehabilitation programs in primary care settings, reporting favorable outcomes in exercise capacity, quality of life, and dyspnea symptoms [58,59]. Our feasibility trial, conducted in a real-world setting, involved app-guided rehabilitation without provider education or guidance. Despite these limitations, we observed improvements in the CAT score and mMRC dyspnea scale, supporting the feasibility of app-based pulmonary rehabilitation in primary care, where center-based programs are generally unavailable.

At an Australian referral center, the referral rate for pulmonary rehabilitation from primary care clinics was notably low—only 6% [60]. This likely reflects limited awareness and understanding of pulmonary rehabilitation among both patients and health care providers in primary care settings [61]. Considering the challenges many patients face in attending regular sessions at tertiary hospitals, as well as the impracticality of tertiary centers accommodating all eligible patients, there is a clear need to implement home-based rehabilitation programs. Using tools such as smartphone apps could enable continuous management of patients within primary care settings.

### Strengths, Limitations, and Future Perspectives

This study has several notable strengths with innovative contributions to the field of digital pulmonary rehabilitation. First, unlike earlier mobile health interventions that targeted single diseases such as COPD or relied on simple video-based exercise guidance, the app offered integrated multidimensional components, incorporating exercise programs, disease education, medication management, social support, and a health data diary, in a single smartphone platform. Particularly, exercise levels could be adjusted according to exercise performance records. The app included social support features that allowed users to see their ranking by comparing their exercise records with those of other users, as well as send and receive encouraging messages with one another. Second, the program was validated not only in a tertiary hospital setting but also through a feasibility trial in primary care clinics, highlighting its scalability and adaptability for community-level implementation. Third, it was quite a novel trial to incorporate an administrator web system that enabled clinicians to monitor adherence and performance data in real time, facilitating a feedback loop between patients and health care providers. It demonstrated the potential of the app as a foundation for a medical information-sharing platform to support patient referrals and transfers. Fourth, this was the first cost-utility analysis of smartphone-based pulmonary rehabilitation conducted in Korea, which may provide health economic evidence to support potential coverage under the National Health Insurance system. Clinically, this integrated digital rehabilitation model can complement or substitute traditional center-based rehabilitation, expanding access for older adults and patients with limited mobility. Together, these innovations demonstrate a practical and evidence-based framework for integrating smartphone-based pulmonary rehabilitation into routine clinical and public health practice.

This study has several limitations. First, while participants showed significant improvements in physical activity according to the IPAQ questionnaire, their correlations to objective measurements are limited [62,63]. Participants’ follow-up VO_2_ max was also lower than that at baseline. Many follow-up tests occurred during a particularly hot summer, which limited participants’ maximal exercise capacity during cardiopulmonary tests, especially as they had outpatient visits scheduled immediately afterward. Additionally, some participants, having experienced fatigue during the baseline test, did not reach their maximal exercise capacity at follow-up. In the control group, 2 participants underwent the 6-minute walk test instead of the cardiopulmonary exercise test due to severely impaired lung function, in accordance with the study protocol. The IPAQ questionnaire was administered during the cardiopulmonary exercise test, so these values were missing for those 2 participants. Although some missing data (IPAQ and DL_CO_) were observed at the follow-up time point, the Little’s test indicated that these data were missing at random. During the feasibility trial, we also noted that participants’ 6-minute walk distance increased by only 5 m after the rehabilitation. Future studies should develop a more refined exercise test protocol to address these challenges. Second, this study did not show statistically significant improvement in the EQ-5D-5L and HINT-8 indices. Studies with larger samples may reveal quality-of-life improvements for patients with chronic respiratory diseases. Third, this study found no improvement in hand grip strength or limb muscle mass in the intervention group, despite providing muscle exercises and nutritional supplements. Although we provided protein supplements of 10 g/day, this amount was far below the recommended daily protein intake for adults of 0.8-1.2 g/kg, so we believe the observed benefits were mainly driven by the app design itself. Future research should consider more tailored exercise programs and nutritional support to enhance physical parameters. Finally, due to several practical constraints, we were unable to set the control group as traditional face-to-face (center-based) pulmonary rehabilitation, which warrants caution when interpreting the findings of this study.

### Conclusions

In the randomized controlled trial, the smartphone-based pulmonary rehabilitation program improved clinical outcomes, including quality of life, physical activity, and dyspnea, in patients with chronic respiratory diseases. Although physically active and program-compliant participants showed significant clinical improvements, the fact that fewer than half of the participants demonstrated good compliance highlights the need for more robust strategies to enhance adherence in future programs. Additionally, while our feasibility trial demonstrated the potential for rehabilitation programs for older adults with chronic respiratory diseases to be implemented in primary health care settings, further research and development will be necessary to achieve this in practice.

## Appendix

Multimedia Appendix 1 CONSORT-eHEALTH checklist (V 1.6.1).

Multimedia Appendix 2 Supplementary tables and figures.

## Abbreviations

CAT: COPD assessment test
COPD: chronic obstructive pulmonary disease
DLCO: diffusing capacity for carbon monoxide
FEV1: forced expiratory volume in one second
FVC: forced vital capacity
HINT-8: Health-related Quality of Life Instrument with 8 Items
IPAQ: International Physical Activity Questionnaire
mMRC: modified Medical Research Council
QALY: quality-adjusted life year
VO2 max: maximal oxygen consumption

## Data Availability

The datasets used and analyzed during the current study are available from the corresponding author upon reasonable request.
