# Supplementary material for: Clinical Efficacy of Smartphone App–Based Pulmonary Rehabilitation in Chronic Respiratory Diseases: Randomized Controlled and Feasibility Trials
Source: J Med Internet Res. 2025 Nov 28;27:e76801. doi: 10.2196/76801 (PMC12701351; doi:10.2196/76801)

**Table S1.** Comparison of clinical outcomes of participants between the intervention and control group (intention to treat analysis)

|  | Total  (n = 70) | Intervention  (n = 46) | Control  (n = 24) | *P* value | *U* value |
| --- | --- | --- | --- | --- | --- |
| Baseline |  |  |  |  |  |
| *VO_2_ max (ml/kg/min, n = 68) | 15.0 [11.5–18.0] | 15.5 [12.6–18.7] | 13.4 [9.0–16.1] | 0.090 | 376.50 |
| CAT score | 14.5 [8.0–19.0] | 16.5 [9.0–20.0] | 11.0 [7.5–17.0] | 0.074 | 408.00 |
| IPAQ (n = 68) | 693.0 [23.1–1498.5] | 742.5 [23.1–1605.0] | 445.5 [11.6–1188.0] | 0.282 | 424.00 |
| mMRC dyspnea scale | 1.0 [1.0–2.0] | 1.0 [1.0–2.0] | 2.0 [1.0–2.0] | 0.631 | 516.50 |
| EQ-5D-5L index | 0.816 [0.750–0.862] | 0.811 [0.740–0.858] | 0.822 [0.777–1.000] | 0.362 | 478.50 |
| HINT-8 index | 0.805 [0.750–0.860] | 0.796 [0.733–0.859] | 0.811 [0.784–0.863] | 0.173 | 442.00 |
| FEV1 (%predicted) | 51.0 [41.0–66.0] | 56.0 [44.0–67.0] | 49.0 [38.5–56.0] | 0.156 | 437.50 |
| FVC (%predicted) | 73.0 [65.0–86.0] | 74.5 [65.0–86.0] | 72.0 [64.0–86.0] | 0.473 | 494.00 |
| DL_CO_ (%predicted) | 56.5 [45.0–67.0] | 57.5 [44.0–67.0] | 50.0 [45.0–65.0] | 0.512 | 499.00 |
| Hand grip strength (kg) | 32.9 [25.7–39.8] | 34.0 [28.1–41.3] | 27.9 [21.5–37.1] | 0.082 | 411.50 |
| Limb muscle mass (kg) |  |  |  |  |  |
| Upper limb | 4.9 [3.5–5.7] | 5.0 [4.0–6.0] | 4.3 [3.3–5.5] | 0.051 | 394.50 |
| Lower limb | 15.0 [12.4–16.6] | 15.6 [13.1–17.0] | 14.5 [10.8–15.6] | 0.047 | 391.50 |
| Follow-up |  |  |  |  |  |
| *VO_2_ max (ml/kg/min, n = 68) | 12.8 [11.0–16.1] | 13.4 [10.5–17.8] | 12.7 [11.3–13.7] | 0.376 | 438.50 |
| CAT score | 8.0 [5.0–17.0] | 7.5 [4.0–16.0] | 10.0 [6.5–18.5] | 0.086 | 413.50 |
| IPAQ (n = 65) | 1386.0 [859.1–2772.0] | 1477.5 [1137.5–3012.0] | 1164.0 [618.8–2205.0] | 0.068 | 332.00 |
| mMRC dyspnea scale | 1.0 [1.0–2.0] | 1.0 [1.0–2.0] | 2.0 [1.0–2.0] | 0.021 | 389.00 |
| EQ-5D-5L index | 0.862 [0.783–1.000] | 0.871 [0.787–1.000] | 0.829 [0.768–1.000] | 0.387 | 483.50 |
| HINT-8 index | 0.819 [0.751–0.876] | 0.825 [0.763–0.882] | 0.795 [0.728–0.848] | 0.136 | 431.50 |
| FEV1 (%predicted) | 50.0 [40.0–68.0] | 58.0 [42.0–69.0] | 45.0 [37.5–54.5] | 0.096 | 417.50 |
| FVC (%predicted) | 73.0 [67.0–86.0] | 75.5 [69.0–86.0] | 71.5 [63.0–86.0] | 0.232 | 455.50 |
| DL_CO_ (%predicted, n = 69) | 60.0 [48.8–71.0] | 62.0 [51.0–71.0] | 57.0 [48.0–64.8] | 0.353 | 456.00 |
| Hand grip strength (kg) | 32.7 [23.3–40.2] | 33.3 [27.8–41.8] | 24.0 [20.6–37.0] | 0.049 | 393.00 |
| Limb muscle mass (kg) |  |  |  |  |  |
| Upper limb | 4.7 [3.7–5.9] | 4.9 [3.8–6.0] | 4.3 [3.5–5.5] | 0.136 | 431.50 |
| Lower limb | 14.9 [12.4–16.6] | 15.4 [13.5–16.9] | 14.4 [10.8–16.0] | 0.074 | 407.50 |

Data are presented as median [interquartile range].

*VO_2_ max was measured using a cardiopulmonary exercise test.

VO_2_ max, maximal oxygen consumption; CAT, chronic obstructive lung disease assessment test; mMRC, mMRC Modified Medical Research Council; IPAQ, International Physical Activity Questionnaire; EQ-5D-5L, Euro-QoL 5-Dimension 5-Level; HINT-8, Health-related Quality of Life Instrument with 8 Items; FEV1, forced expiratory volume in one second; FVC, forced vital capacity; DL_CO_, diffusing capacity for carbon monoxide

**Table S2.** Comparison of clinical outcomes of participants between the baseline and follow-up (intention to treat analysis)

|  | Baseline | Follow-up | *P* value |
| --- | --- | --- | --- |
| Intervention (n = 46) |  |  |  |
| *VO_2_ max (ml/kg/min) | 15.5 [12.6–18.7] | 13.4 [10.5–17.8] | 0.031 |
| CAT score | 16.5 [9.0–20.0] | 7.5 [4.0–16.0] | < 0.001 |
| IPAQ (n = 44) | 742.5 [23.1–1605.0] | 1477.5 [1137.5–3012.0] | < 0.001 |
| mMRC dyspnea scale | 1.0 [1.0–2.0] | 1.0 [1.0–2.0] | 0.004 |
| EQ-5D-5L index | 0.811 [0.740–0.858] | 0.871 [0.787–1.000] | < 0.001 |
| HINT-8 index | 0.796 [0.733–0.859] | 0.825 [0.763–0.882] | < 0.001 |
| FEV1 (%predicted) | 56.0 [44.0–67.0] | 58.0 [42.0–69.0] | 0.299 |
| FVC (%predicted) | 75.0 [65.0–86.0] | 75.5 [69.0–86.0] | 0.019 |
| DL_CO_ (%predicted) | 57.5 [44.0–67.0] | 62.0 [51.0–71.0] | 0.064 |
| Hand grip strength (kg) | 34.0 [28.1–41.3] | 33.3 [27.8–41.8] | 0.076 |
| Limb muscle mass (kg) |  |  |  |
| Upper limb | 5.0 [4.0–6.0] | 4.9 [3.8–6.0] | 0.532 |
| Lower limb | 15.6 [13.1–17.0] | 15.4 [13.5–16.9] | 0.404 |
| Control (n = 24) |  |  |  |
| *VO_2_ max (ml/kg/min, n = 22) | 13.4 [9.0–16.1] | 12.7 [11.3–13.7] | 0.446 |
| CAT score | 11.0 [7.5–17.0] | 10.0 [6.5–18.5] | 0.782 |
| IPAQ (n = 21) | 198.0 [11.6–1237.5] | 1164.0 [618.8–2205.0] | 0.005 |
| mMRC dyspnea scale | 2.0 [1.0–2.0] | 2.0 [1.0–2.0] | 0.739 |
| EQ-5D-5L index | 0.822 [0.777–1.000] | 0.829 [0.768–1.000] | 0.257 |
| HINT-8 index | 0.811 [0.784–0.863] | 0.795 [0.728–0.848] | 0.085 |
| FEV1 (%predicted) | 49.0 [38.5–56.0] | 45.0 [37.5–54.5] | 0.464 |
| FVC (%predicted) | 72.0 [64.0–86.0] | 71.5 [63.0–86.0] | 0.779 |
| DL_CO_ (%predicted, n = 23) | 50.0 [45.0–65.0] | 57.0 [48.0–64.8] | 0.167 |
| Hand grip strength (kg) | 27.9 [21.5–37.1] | 24.0 [20.6–37.0] | 0.290 |
| Limb muscle mass (kg) |  |  |  |
| Upper limb | 4.3 [3.3–5.5] | 4.3 [3.5–5.5] | 0.117 |
| Lower limb | 14.5 [10.8–15.6] | 14.4 [10.8–16.0] | 0.670 |

Data are presented as median [interquartile range].

*VO_2_ max was measured using a cardiopulmonary exercise test.

VO_2_ max, maximal oxygen consumption; CAT, chronic obstructive lung disease assessment test; mMRC, mMRC Modified Medical Research Council; IPAQ, International Physical Activity Questionnaire; EQ-5D-5L, Euro-QoL 5-Dimension 5-Level; HINT-8, Health-related Quality of Life Instrument with 8 Items; FEV1, forced expiratory volume in one second; FVC, forced vital capacity; DL_CO_, diffusing capacity for carbon monoxide

**Table S3.** Comparison of clinical outcomes of participants between the intervention (initially active, IPAQ > 1000) and control group. (Subgroup analysis)

|  | Total  (n = 43) | Intervention  (n = 19) | Control  (n = 24) | *P* value | *U* value |
| --- | --- | --- | --- | --- | --- |
| Baseline |  |  |  |  |  |
| *VO_2_ max (ml/kg/min, n = 41) | 14.8 [11.2–17.9] | 15.6 [13.4–18.1] | 13.4 [9.0–16.1] | 0.108 | 147.50 |
| CAT score | 12.0 [7.3–18.0] | 17.0 [7.5–19.0] | 11.0 [7.5–17.0] | 0.203 | 176.00 |
| IPAQ (n = 41) | 1386.0 [198.0–2079.0] | 1836.0 [1485.0–3297.0] | 445.5 [11.6–1188.0] | < 0.001 | 57.00 |
| mMRC dyspnea scale | 2.0 [1.0–2.0] | 1.0 [1.0–2.8] | 2.0 [1.0–2.0] | 0.916 | 224.00 |
| EQ-5D-5L index | 0.816 [0.763–0.862] | 0.804 [0.723–0.859] | 0.822 [0.777–1.000] | 0.461 | 198.00 |
| HINT-8 index | 0.807 [0.774–0.862] | 0.806 [0.757–0.856] | 0.811 [0.784–0.863] | 0.385 | 192.50 |
| FEV1 (%predicted) | 48.0 [41.3–58.0] | 48.0 [44.3–62.8] | 49.0 [38.5–56.0] | 0.557 | 204.00 |
| FVC (%predicted) | 73.0 [63.0–89.0] | 75.0 [63.0–90.5] | 72.0 [64.0–86.0] | 0.470 | 198.50 |
| DL_CO_ (%predicted) | 58.0 [45.5–67.5] | 62.0 [55.3–68.3] | 50.0 [45.0–65.0] | 0.152 | 169.50 |
| Hand grip strength (kg) | 34.1 [25.8–40.8] | 37.1 [31.3–44.7] | 27.9 [21.5–37.1] | 0.014 | 127.50 |
| Limb muscle mass (kg) |  |  |  |  |  |
| Upper limb | 5.1 [3.5–5.8] | 5.7 [4.7–6.1] | 4.3 [3.3–5.5] | 0.004 | 110.00 |
| Lower limb | 14.9 [12.0–16.9] | 16.4 [13.2–17.9] | 14.5 [10.8–15.6] | 0.020 | 133.00 |
| Follow-up |  |  |  |  |  |
| *VO_2_ max (ml/kg/min, n = 41) | 12.9 [11.7–15.2] | 14.3 [12.0–19.1] | 12.7 [11.3–13.7] | 0.050 | 134.00 |
| CAT score | 7.0 [5.0–16.8] | 5.0 [3.3–10.0] | 10.0 [6.5–18.5] | 0.005 | 114.50 |
| IPAQ (n = 39) | 1386.0 [825.4–2965.5] | 2799.0 [1386.0–3324.0] | 1164.0 [618.8–2205.0] | 0.009 | 96.00 |
| mMRC dyspnea scale | 1.0 [1.0–2.0] | 1.0 [1.0–1.0] | 2.0 [1.0–2.0] | 0.034 | 151.50 |
| EQ-5D-5L index | 0.862 [0.783–1.000] | 0.871 [0.837–1.000] | 0.829 [0.768–1.000] | 0.114 | 165.50 |
| HINT-8 index | 0.821 [0.744–0.876] | 0.843 [0.805–0.906] | 0.795 [0.728–0.848] | 0.030 | 139.50 |
| FEV1 (%predicted) | 48.0 [39.3–57.5] | 49.0 [42.3–62.5] | 45.0 [37.5–54.5] | 0.316 | 187.00 |
| FVC (%predicted) | 72.0 [65.3–88.0] | 72.0 [66.3–88.8] | 71.5 [63.0–86.0] | 0.501 | 200.50 |
| DL_CO_ (%predicted, n = 42) | 60.0 [50.0–68.0] | 63.0 [53.3–71.0] | 57.0 [48.0–64.8] | 0.136 | 159.50 |
| Hand grip strength (kg) | 32.7 [22.8–39.5] | 34.2 [31.8–42.9] | 24.0 [20.6–37.0] | 0.018 | 131.00 |
| Limb muscle mass (kg) |  |  |  |  |  |
| Upper limb | 5.0 [3.7–6.0] | 5.9 [4.2–6.6] | 4.3 [3.5–5.5] | 0.014 | 128.00 |
| Lower limb | 15.4 [12.1–16.9] | 16.5 [13.7–17.5] | 14.4 [10.8–16.0] | 0.018 | 132.00 |

Data are presented as median [interquartile range].

*VO_2_ max was measured using a cardiopulmonary exercise test.

VO_2_ max, maximal oxygen consumption; CAT, chronic obstructive lung disease assessment test; mMRC, mMRC Modified Medical Research Council; IPAQ, International Physical Activity Questionnaire; EQ-5D-5L, Euro-QoL 5-Dimension 5-Level; HINT-8, Health-related Quality of Life Instrument with 8 Items; FEV1, forced expiratory volume in one second; FVC, forced vital capacity; DL_CO_, diffusing capacity for carbon monoxide

**Table S4.** Comparison of clinical outcomes of participants (the intervention group, initially active, IPAQ > 1000) between the baseline and follow-up

|  | Baseline | Follow-up | *P* value |
| --- | --- | --- | --- |
| Intervention (n = 19) |  |  |  |
| *VO_2_ max (ml/kg/min) | 15.6 [13.4–18.1] | 14.3 [12.0–19.1] | 0.421 |
| CAT score | 17.0 [7.5–19.0] | 5.0 [3.3–10.0] | < 0.001 |
| IPAQ (n = 18) | 1809.0 [1485.0–3180.0] | 2799.0 [1386.0–3324.0] | 0.523 |
| mMRC dyspnea scale | 1.0 [1.0–2.8] | 1.0 [1.0–1.0] | 0.011 |
| EQ-5D-5L index | 0.804 [0.723–0.859] | 0.871 [0.837–1.000] | < 0.001 |
| HINT-8 index | 0.806 [0.757–0.856] | 0.843 [0.805–0.906] | 0.001 |
| FEV1 (%predicted) | 48.0 [44.3–62.8] | 49.0 [42.3–62.5] | 0.887 |
| FVC (%predicted) | 75.0 [63.0–90.5] | 72.0 [66.3–88.8] | 0.614 |
| DL_CO_ (%predicted) | 62.0 [55.3–68.3] | 63.0 [53.3–71.0] | 0.420 |
| Hand grip strength (kg) | 37.1 [31.3–44.6] | 34.2 [31.7–42.9] | 0.227 |
| Limb muscle mass (kg) |  |  |  |
| Upper limb | 5.7 [4.7–6.1] | 5.9 [4.2–6.6] | 0.840 |
| Lower limb | 16.4 [13.2–17.9] | 16.5 [13.7–17.5] | 0.695 |

Data are presented as median [interquartile range].

*VO_2_ max was measured using a cardiopulmonary exercise test.

VO_2_ max, maximal oxygen consumption; CAT, chronic obstructive lung disease assessment test; mMRC, mMRC Modified Medical Research Council; IPAQ, International Physical Activity Questionnaire; EQ-5D-5L, Euro-QoL 5-Dimension 5-Level; HINT-8, Health-related Quality of Life Instrument with 8 Items; FEV1, forced expiratory volume in one second; FVC, forced vital capacity; DL_CO_, diffusing capacity for carbon monoxide

**Table S5.** Comparison of clinical outcomes of participants between the intervention (compliance > 50%) and control group. – subgroup analysis

|  | Total  (n = 41) | Intervention  (n = 17) | Control  (n = 24) | *P* value | *U* value |
| --- | --- | --- | --- | --- | --- |
| Baseline |  |  |  |  |  |
| *VO_2_ max (ml/kg/min, n = 39) | 15.4 [10.5–20.0] | 17.9 [13.7–23.5] | 13.4 [9.0–16.1] | 0.024 | 107.50 |
| CAT score | 14.0 [9.0–18.3] | 17.0 [11.8–20.8] | 11.0 [7.5–17.0] | 0.023 | 118.00 |
| IPAQ (n = 39) | 792.0 [24.8–1611.0] | 792.0 [302.8–2258.3] | 445.5 [11.6–1188.0] | 0.161 | 137.50 |
| mMRC dyspnea scale | 2.0 [1.0–2.0] | 2.0 [1.0–2.3] | 2.0 [1.0–2.0] | 0.966 | 202.50 |
| EQ-5D-5L index | 0.816 [0.746–0.862] | 0.792 [0.732–0.821] | 0.822 [0.777–1.000] | 0.188 | 154.50 |
| HINT-8 index | 0.806 [0.751–0.862] | 0.792 [0.732–0.836] | 0.811 [0.784–0.863] | 0.153 | 150.00 |
| FEV1 (%predicted) | 50.0 [41.5–60.8] | 53.0 [44.0–68.0] | 49.0 [38.5–56.0] | 0.272 | 162.50 |
| FVC (%predicted) | 73.0 [62.5–88.3] | 78.0 [62.5–88.3] | 72.0 [64.0–86.0] | 0.443 | 175.00 |
| DL_CO_ (%predicted) | 58.0 [45.0–67.3] | 62.0 [52.8–67.5] | 50.0 [45.0–65.0] | 0.327 | 167.00 |
| Hand grip strength (kg) | 31.8 [25.1–39.3] | 34.1 [29.8–42.0] | 27.9 [21.5–37.1] | 0.098 | 141.50 |
| Limb muscle mass (kg) |  |  |  |  |  |
| Upper limb | 4.7 [3.5–5.5] | 5.0 [4.3–5.5] | 4.3 [3.3–5.5] | 0.204 | 156.00 |
| Lower limb | 14.5 [12.4–15.9] | 14.9 [13.6–16.4] | 14.5 [10.8–15.6] | 0.199 | 155.50 |
| Follow-up |  |  |  |  |  |
| *VO_2_ max (ml/kg/min, n = 39) | 12.9 [11.4–16.2] | 16.2 [12.0–21.6] | 12.7 [11.3–13.7] | 0.040 | 114.50 |
| CAT score | 10.0 [5.0–17.3] | 10.0 [3.0–16.3] | 10.0 [6.5–18.5] | 0.272 | 162.50 |
| IPAQ (n = 37) | 1386.0 [859.1–2772.0] | 2359.5 [1270.5–3163.5] | 1164.0 [618.8–2205.0] | 0.025 | 95.00 |
| mMRC dyspnea scale | 1.0 [1.0–2.0] | 1.0 [1.0–2.0] | 2.0 [1.0–2.0] | 0.145 | 154.50 |
| EQ-5D-5L index | 0.829 [0.772–1.000] | 0.846 [0.772–0.871] | 0.829 [0.768–1.000] | 0.779 | 193.50 |
| HINT-8 index | 0.804 [0.746–0.856] | 0.822 [0.782–0.861] | 0.795 [0.728–0.848] | 0.272 | 162.50 |
| FEV1 (%predicted) | 48.0 [39.8–67.5] | 58.0 [45.8–70.0] | 45.0 [37.5–54.5] | 0.118 | 145.00 |
| FVC (%predicted) | 73.0 [64.8–88.5] | 79.0 [69.0–90.5] | 71.5 [63.0–86.0] | 0.302 | 165.00 |
| DL_CO_ (%predicted, n = 40) | 61.0 [48.5–70.5] | 67.0 [57.8–71.3] | 57.0 [48.0–64.8] | 0.106 | 136.50 |
| Hand grip strength (kg) | 31.4 [22.5–38.5] | 35.1 [29.3–42.4] | 24.0 [20.6–37.0] | 0.053 | 131.00 |
| Limb muscle mass (kg) |  |  |  |  |  |
| Upper limb | 4.6 [3.6–5.6] | 4.6 [4.1–5.6] | 4.3 [3.5–5.5] | 0.354 | 169.00 |
| Lower limb | 14.5 [12.3–16.0] | 14.5 [13.5–16.4] | 14.4 [10.8–16.0] | 0.264 | 161.00 |

Data are presented as median [interquartile range].

*VO_2_ max was measured using a cardiopulmonary exercise test.

VO_2_ max, maximal oxygen consumption; CAT, chronic obstructive lung disease assessment test; mMRC, mMRC Modified Medical Research Council; IPAQ, International Physical Activity Questionnaire; EQ-5D-5L, Euro-QoL 5-Dimension 5-Level; HINT-8, Health-related Quality of Life Instrument with 8 Items; FEV1, forced expiratory volume in one second; FVC, forced vital capacity; DL_CO_, diffusing capacity for carbon monoxide

**Table S6.** Comparison of clinical outcomes of participants (the intervention group, compliance > 50%, between the baseline and follow-up

|  | Baseline | Follow-up | *P* value |
| --- | --- | --- | --- |
| Intervention (n = 17) |  |  |  |
| *VO_2_ max (ml/kg/min) | 17.9 [13.7–23.5] | 14.5 [13.5–16.4] | 0.071 |
| CAT score | 17.0 [11.8–20.8] | 10.0 [3.0–16.3] | 0.004 |
| IPAQ (n = 16) | 792.0 [209.6–1908.0] | 2359.5 [1270.5–3163.5] | 0.013 |
| mMRC dyspnea scale | 2.0 [1.0–2.3] | 1.0 [1.0–2.0] | 0.083 |
| EQ-5D-5L index | 0.792 [0.732–0.821] | 0.846 [0.772–0.871] | 0.009 |
| HINT-8 index | 0.792 [0.732–0.836] | 0.822 [0.782–0.861] | 0.034 |
| FEV1 (%predicted) | 53.0 [44.0–68.0] | 58.0 [45.8–70.0] | 0.346 |
| FVC (%predicted) | 78.0 [62.5–88.3] | 79.0 [69.0–90.5] | 0.079 |
| DL_CO_ (%predicted) | 62.0 [52.8–67.5] | 67.0 [57.8–71.3] | 0.038 |
| Hand grip strength (kg) | 34.1 [29.8–42.0] | 35.1 [29.3–42.4] | 0.813 |
| Limb muscle mass (kg) |  |  |  |
| Upper limb | 5.0 [4.3–5.5] | 4.6 [4.1–5.6] | 0.313 |
| Lower limb | 14.9 [13.6–16.4] | 14.5 [13.5–16.4] | 0.517 |

Data are presented as median [interquartile range].

*VO_2_ max was measured using a cardiopulmonary exercise test.

VO_2_ max, maximal oxygen consumption; CAT, chronic obstructive lung disease assessment test; mMRC, mMRC Modified Medical Research Council; IPAQ, International Physical Activity Questionnaire; EQ-5D-5L, Euro-QoL 5-Dimension 5-Level; HINT-8, Health-related Quality of Life Instrument with 8 Items; FEV1, forced expiratory volume in one second; FVC, forced vital capacity; DL_CO_, diffusing capacity for carbon monoxide

**Table S7.** Application service evaluation questionnaire

| Question | n = 43 |
| --- | --- |
| How easy and convenient was the application service provided to you? |  |
| Very easy | 23 (53.5%) |
| Easy | 12 (27.9%) |
| So so | 5 (11.6%) |
| Difficult | 2 (4.7%) |
| Very difficult | 1 (2.3%) |
| How helpful was the application service to improv your dyspnea symptom? |  |
| Very helpful | 12 (27.9%) |
| Helpful | 15 (34.9%) |
| So so | 11 (25.6%) |
| Not helpful | 1 (2.3%) |
| Not helpful at all | 4 (9.3%) |
| Do you want to use the application service if it is commercialized? |  |
| Yes | 34 (79.1%) |
| No | 9 (20.9%) |
| If yes, why do you want to use it? |  |
| I can exercise with the app, although I do not visit hospital. | 0 (0%) |
| I can get a reward if I exercise. | 0 (0%) |
| I can check how much I exercise. | 14 (41.2%) |
| The app suggests exercise suitable to my condition. | 15 (44.1%) |
| I think that the app is easier and more convenient than other apps. | 5 (14.7%) |
| To use the app is more convenient than to visit hospital. | 0 (0%) |
| What was the most attractive point of the application service? |  |
| It is good to have a partner to exercise with. | 1 (2.3%) |
| It is good to get a reward after I exercise. | 1 (2.3%) |
| It is good to be able to check how much I have exercised. | 16 (37.2%) |
| It is good that the application suggests exercise suitable to my condition. | 18 (41.9%) |
| It is good that the app is easier to use and understand than other apps. | 7 (16.3%) |

Data are presented as count (%).

**Table S8.** Participants of feasibility trial

|  | Inclusion | Drop-out | Follow-up |
| --- | --- | --- | --- |
| Maseok Asan Clinic Internal Medicine | 11 | 0 | 11 |
| Seoul Eco Internal Medical Clinic | 3 | 2 | 1 |
| Seoul Comfort Medical Clinic | 10 | 4 | 6 |
| Good Breath Internal Medicine Clinic | 8 | 2 | 6 |
| Total | 32 | 8 | 24 |

**Table S9.** Baseline characteristics of participants in a feasibility trial

|  | Total (n = 24) |
| --- | --- |
| Age (years) | 62.5 [57.0–69.0] |
| Male sex | 14 (58.3) |
| Body weight (kg) | 65.0 [61.5–77.0] |
| Height (cm) | 165.0 [157.0–173.0] |
| BMI (kg/m^2^) | 26.1 [23.5–27.7] |
| Ever-smoker | 13 (54.2) |
| Respiratory disease |  |
| Obstructive | 20 (83.3) |
| Bronchiectasis | 0 (0.0) |
| Restrictive | 4 (16.7) |
| Comorbidities |  |
| Diabetes mellitus | 3 (12.5) |
| Hypertension | 14 (58.3) |
| Dyslipidemia | 14 (58.3) |
| Malignancy | 1 (4.2) |
| Lung resection | 1 (4.2) |

Data are presented as median [interquartile range] or count (%), unless otherwise indicated.

BMI, body mass index

**Table S10.** Clinical outcomes of participants in a feasibility trial

|  | Baseline | Follow-up | *P* value |
| --- | --- | --- | --- |
| 6-minute walk distance (m) | 436.5 [403.0–478.0] | 441.5 [326.0–487.0] | 0.265 |
| CAT score | 8.5 [6.0–18.0] | 5.0 [2.0–7.5] | < 0.001 |
| mMRC dyspnea scale | 1.0 [1.0–1.0] | 1.0 [0.0–1.0] | 0.007 |
| FEV1 (%predicted) | 70.0 [61.5–77.0] | 68.0 [58.0–76.5] | 0.262 |
| FVC (%predicted) | 73.5 [69.5–89.0] | 69.0 [65.0–83.0] | 0.011 |

Data are presented as median [interquartile range].

CAT, chronic obstructive lung disease assessment test; mMRC, mMRC Modified Medical Research Council; FEV1, forced expiratory volume in one second; FVC, forced vital capacity

**Figure S1.** Screenshots of the “SENIORS” application (A) Exercise schedule (B) Walking exercise (C) Exercise record (D) Exercise ranking (E) Reward


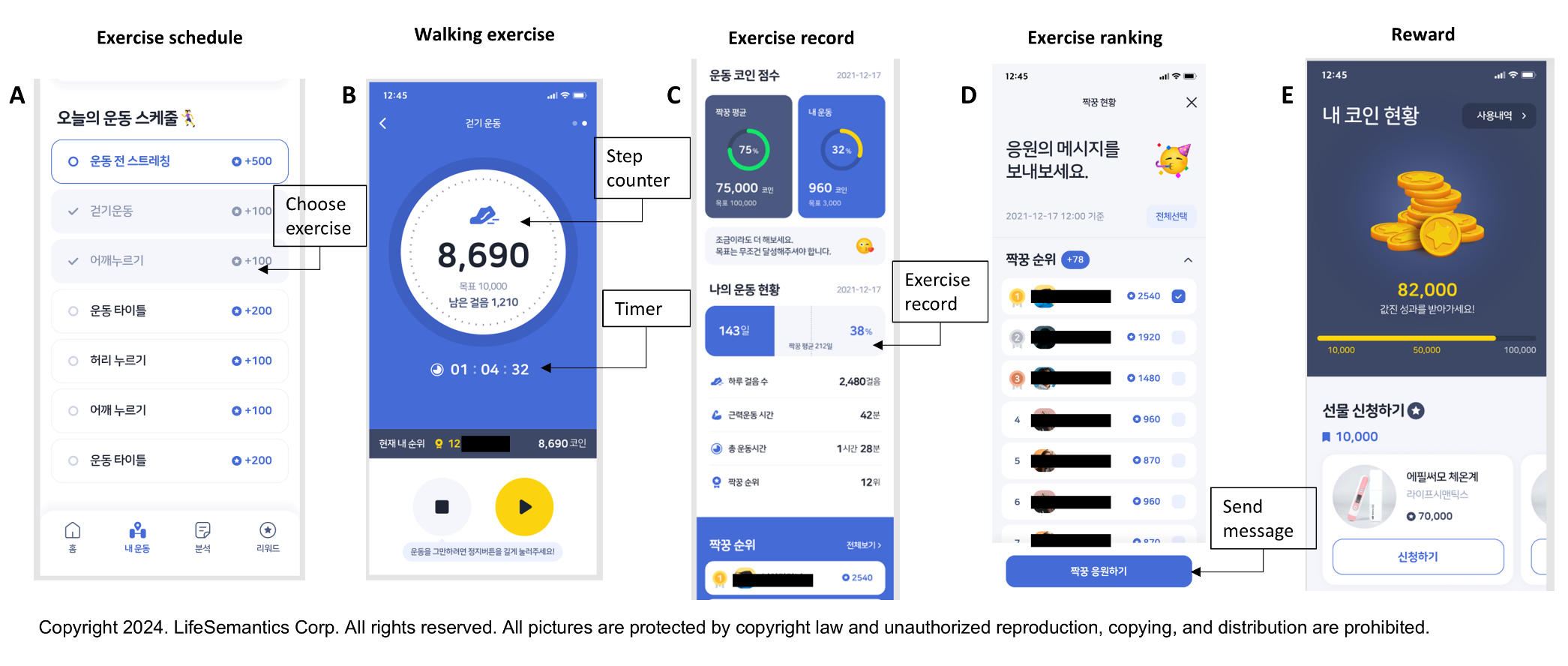


**Figure S2.** Change in COPD assessment test score of the intervention group


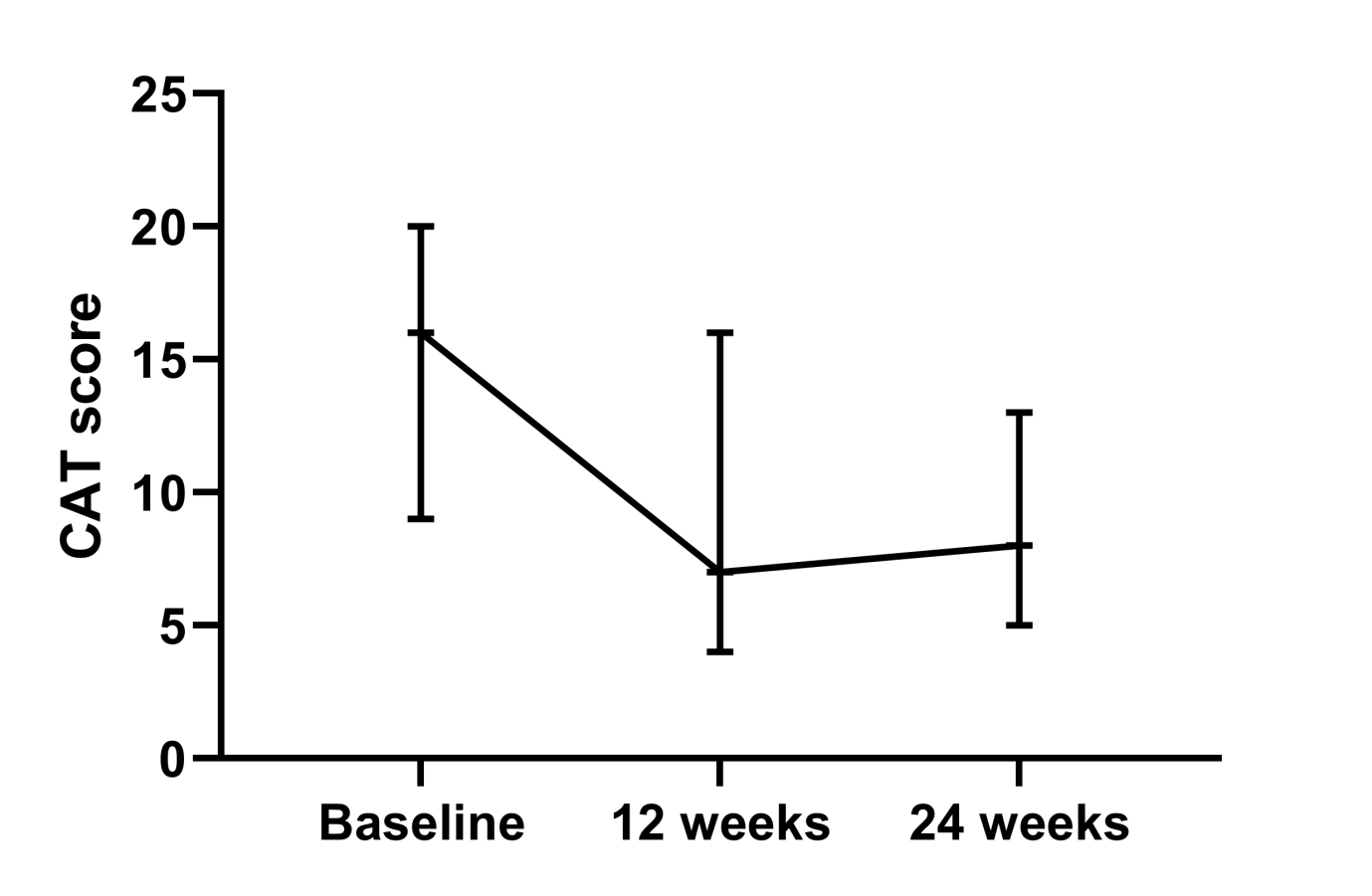

Supplement: Multimedia Appendix 2 [file jmir_v27i1e76801_app2.docx]
